# *α*-Tocopheryl succinate and TRAIL selectively synergise in induction of apoptosis in human malignant mesothelioma cells

**DOI:** 10.1038/sj.bjc.6601707

**Published:** 2004-03-23

**Authors:** M Tomasetti, M R Rippo, R Alleva, S Moretti, L Andera, J Neuzil, A Procopio

**Affiliations:** 1 Department of Molecular Pathology and Innovative Therapies, Polytechnic University of Marche, 60131 Ancona, Italy; 2 Department of Anesthesiology, IRCCS Istituti Ortopedici Rizzoli, 40100 Bologna, Italy; 3 Institute of Molecular Genetics, Czech Academy of Sciences, 14000 Prague, Czech Republic; 4 Department of Pathology II, Faculty of Health Sciences, University Hospital, 58183 Linköping, Sweden; 5 School of Health Sciences, Griffith University Gold Coast Campus, Southport, 9726 Queensland, Australia

**Keywords:** *α*-tocopheryl succinate, TRAIL, synergism, apoptosis, malignant mesothelioma

## Abstract

Malignant mesothelioma (MM) is a fatal type of neoplasia with poor therapeutic prognosis, largely due to resistance to apoptosis. We investigated the apoptotic effect of *α*-tocopheryl succinate (*α*-TOS), a strong proapoptotic agent, in combination with the immunological apoptogen TNF-related apoptosis-inducing ligand (TRAIL) on both MM and nonmalignant mesothelial cells, since MM cells show low susceptibility to the clinically intriguing TRAIL. All MM cell lines tested were sensitive to *α*-TOS-induced apoptosis, and exerted high sensitivity to TRAIL in the presence of subapoptotic doses of the vitamin E analogue. Neither TRAIL or *α*-TOS alone or in combination caused apoptosis in nonmalignant mesothelial cells. Isobologram analysis of the cytotoxicity assays revealed a synergistic interaction between the two agents in MM cells and their antagonistic effect in nonmalignant mesothelial cells. TRAIL-induced apoptosis and its augmentation by *α*-TOS were inhibited by the caspase-8 inhibitor Z-IETD-FMK and the pan-caspase inhibitor Z-VAD-FMK. Activation of caspase-8 was required to induce apoptosis, which was amplified by *α*-TOS via cytochrome *c* release following Bid cleavage, with ensuing activation of caspase-9. Enhancement of TRAIL-induced apoptosis in MM cells by *α*-TOS was also associated with upregulation of the TRAIL cognate death receptors DR4 and DR5. Our results show that *α*-TOS and TRAIL act in synergism to kill MM cells via mitochondrial pathway, and are nontoxic to nonmalignant mesothelial cells. These findings are indicative of a novel strategy for treatment of thus far fatal MM.

Human malignant mesothelioma (MM) represents a tumour, which arises from mesothelial cells of the pleura or peritoneal cavities. The pathogenesis of this disease is associated with exposure to asbestos fibres (Mossman *et al*, 1996). Recently, Simian virus-40 (SV-40) has been associated with this malignancy (Carbone *et al*, 1994). The SV-40 oncogenic proteins bind and inactivate cellular p53 (Carbone *et al*, 1997) and the SV-40-positive status negatively affects the prognostic outcome of MM patients (Procopio *et al*, 2000). A typical feature of MM is its resistance to both chemotherapeutic agents and radiation (Houghton, 1999; Tomek *et al*, 2003). The general resistance of MM cells to apoptosis may explain the insensitivity of mesothelioma to therapy (Narasimhan *et al*, 1998), which has been shown for a variety of apoptotic agonists, although its mechanism has not been elucidated (Soini *et al*, 1999).

The immunological proapoptotic agent, tumour necrosis factor-related apoptosis-inducing ligand (TRAIL, Apo2L) induces apoptosis in a wide variety of malignant cells without exhibiting systemic toxicity to normal cells or tissues (French and Tschopp, 1999). The mechanism of TRAIL proapoptotic signalling includes its interaction with two death domain-containing receptors, DR4 (TRAIL-R1) and DR5 (TRAIL-R2). Although DR4 and DR5 are both widely expressed in human tissue, normal cells are usually resistant to TRAIL (Ashkenazi and Dixit, 1998). The reason may be that normal cells often express TRAIL decoy receptors (DcRs), DcR1 (TRAIL-R3) and DcR2 (TRAIL-R4), which compete with the death receptors (DRs) (Ashkenazi and Dixit, 1999). Based on the observations of an increase in DR5 expression by DNA-damaging agents that induce apoptosis in a p53-dependent manner (Wu *et al*, 1997), it has been suggested that TRAIL-dependent signalling may be related to p53 activation (Sheikh *et al*, 1998). Thus, agents capable of upregulating expression of TRAIL DRs are likely to enhance susceptibility of cells to TRAIL. Indeed, chemotherapeutic compounds like doxorubicine, 5-fluorouracil, and CPT-11 showed a synergy in apoptosis induction with TRAIL, and suppressed tumour growth in animal models (Gliniak and Le, 1999; Keane *et al*, 1999). These findings are consistent with the notion that cancer can be treated more efficiently by combination of agents with synergistic/additive activities.

Several groups have shown that a semisynthetic analogue of vitamin E, *α*-tocopheryl succinate (*α*-TOS), is a potent inducer of apoptosis in a variety of malignant cells (Yu *et al*, 1999; Yamamoto *et al*, 2000; Neuzil *et al*, 2001a), while being largely nontoxic to normal cells and tissues (Neuzil *et al*, 2001a; Weber *et al*, 2002). The apoptotic effect of *α*-TOS involves lysosomal and mitochondrial destabilisation as well as caspase-3 activation (Alleva *et al*, 2001; Neuzil *et al*, 2001b). *α*-Tocopheryl succinate-induced apoptosis may be amplified by protein kinase C (PKC) inhibition (Neuzil *et al*, 2001b). Recently, *α*-TOS has been shown to exert antineoplastic activity and to promote tumour dormancy in preclinical models (Malafa and Neitzel, 2000; Weber *et al*, 2002). Finally, *α*-TOS has been reported to sensitise resistant breast cancer cells to Fas killing (Yu *et al*, 1999) as well as to cooperate with TRAIL in apoptosis induction in colon cancer cells and in colon carcinoma growth suppression (Weber *et al*, 2002).

Here we have investigated the effect of *α*-TOS and TRAIL in MM cells to test their potential ability to cooperatively induce apoptosis. We show that MM cells are highly susceptible to *α*-TOS, which synergises with TRAIL. Neither *α*-TOS nor TRAIL alone or in combination showed toxicity to nonmalignant mesothelial cells; rather, the *α*-TOS antagonised the nonmalignant cells to the effects of TRAIL. These findings support the emerging idea of the vitamin E analogue as a novel anticancer agent and/or adjuvant (Neuzil, 2002).

## MATERIALS AND METHODS

### Reagents

*α*-Tocopheryl succinate, annexin V-FITC, low melting point agarose, agarose, (3-4,5-dimethylthiazol-2-yl)-2,5-diphenyltetrazolium bromide (MTT), and 4′,6-diamino-2-phenylindole (DAPI) were purchased from Sigma (St Louis, MO, USA). Soluble human recombinant tumour necrosis factor-related apoptosis-inducing ligand (hrTRAIL) was prepared as described elsewhere (Plasilova *et al*, 2002). Anti-DR4, anti-DR5, and anti-DcR1 monoclonal IgG were obtained from Alexis Biochemicals (Lausen, Switzerland), and the anti-DcR2 polyclonal IgG from ProSci (Poway, CA, USA), anticytochrome *c* and anti-Bid IgG were from Pharmigen (San Diego, CA, USA), anticaspase-8 monoclonal IgG from Upstate Biotechnology (Lake Placid, NY, USA), and anticaspase-9 polyclonal IgG from Santa Cruz (Santa Cruz, CA, USA). All primers for RT–PCR were obtained from Sigma Genosys (St Louis, MO, USA). The colorimetric substrates for caspase-8 (Ac-IETD-pNA) and caspase-9 (Ac-LEHD-pNA), the caspase-8 inhibitor (Z-IETD-FMK), and the pan-caspase inhibitor (Z-VAD-FMK) were purchased from Calbiochem (San Diego, CA, USA). Foetal Bovine Serum (FBS) was obtained from EuroClone (Paignton, UK). The plasmids harbouring the green fluorescent protein (GFP) and the red fluorescence protein (RFP), pGFP, and pDsRed1-N1, respectively, were obtained from BD Biosciences (Palo Alto, CA, USA).

### Cell culture

The MM-B1 (biphasic), Meso-2 (sarcomatoid), and Ist-Mes (epithelioid) human MM cell lines were used (Pass *et al*, 1995). The nonmalignant mesothelial cell line, Met-5A (ATTC, Rockville, MD, USA), was used as a nonmalignant control. HeLa cells were obtained from ATCC and used as a positive control for the expression of TRAIL receptors. The cells were cultured in the RPMI-1640 medium supplemented with 2 mM L-glutamine, 100 U ml^−1^ penicillin, 100 *μ*g ml^−1^ streptomycin, and 10% FBS.

### Cytotoxicity and isobologram analysis

MM cells were plated in 96-well flat-bottom tissue culture plate at 2.5 × 10^3^ per well. The cells were allowed to attach overnight, and then incubated for 24 h with *α*-TOS and hrTRAIL at ⩽100 *μ*M and ⩽10 *μ*g ml^−1^, respectively. *α*-Tocopheryl succinate was dissolved in ethanol and diluted in complete RPMI-1640 to the final concentration, and was added to cells at 0.1% (v v^−1^) of ethanol. Cell viability were determined using MTT (Carmichael *et al*, 1987). Briefly, following exposure of cells, 10 *μ*l of MTT (5 mg ml^−1^ in PBS) was added, and after incubation for 4 h at 37°C, the medium was removed and combined with 200 *μ*l of 1% SDS. Absorbance was read at 550 nm using an ELISA plate reader and control absorbance was designed as 100%, with percentage of it as a cell survival. Survival curves were generated for *α*-TOS and hrTRAIL, and the IC_50_ values were determined. The effect of combination of the drugs on MM and nonmalignant mesothelial cells was estimated by using the isobologram evaluation (Tsai *et al*, 1989). Briefly, the cells were incubated with both agents added simultaneously to the cell culture medium at different concentrations, and the IC_50_ values for each drug in combination were calculated. Isobolograms were plotted at the individual IC_50_ values.

### Apoptosis detection

#### Annexin V-FITC assay

Apoptosis was quantified using the annexin V-FITC method, which detects phosphatidyl serine (PS) externalised in the early phases of apoptosis (Boersma *et al*, 1996). Briefly, cells were plated at 10^5^ per well in 24-well plates. After an overnight incubation, cells were treated with *α*-TOS (30 *μ*M) or hrTRAIL (50 ng ml^−1^) alone or in combination. Floating and attached cells were collected, washed twice with PBS, resuspended in 0.1 ml binding buffer (10 mM HEPES, 140 mM NaCl, 5 mM CaCl_2_, pH 7.4), incubated for 20 min at room temperature with 2 *μ*l annexin V-FITC, supplemented with 10 *μ*l of propidium iodide (PI) (10 *μ*g ml^−1^), and analysed by flow cytometry (Becton Dickinson, Rutherford, NJ, USA), using channel 1 for annexin V-FITC binding and channel 2 for PI staining.

#### Comet assay

DNA fragmentation was analysed using the neutral comet assay (Kizilian *et al*, 1999). Cells were sandwiched between thin layers of agarose on a microscope slide, lysed at neutral pH, electrophoresed, and stained with a silver dye. During electrophoresis, loops or pieces of DNA migrate away from the nuclear remnant to produce a shape with the visual characteristics of a comet. The comet assay can thus distinguish apoptotic from nonapoptotic cells on the basis of their characteristic DNA fragmentation pattern (Tomasetti *et al*, 2001).

### Western blotting

Cells were treated as indicated and lysed in a buffer containing 1% Nonidet, 5 mM DTT, 150 mM NaCl, 2 mM EDTA, 50 mM Tris-HCl, pH 7.4, and a cocktail of protease inhibitors, and stored at −80°C until analysis. Cell extracts were mixed with the sample buffer containing 12 mM Tris-HCl, pH 6.8, 6% SDS, 10% *β*-mercaptoethanol, 20% glycerol, and 0.03% bromophenol blue. Protein samples (100 *μ*g per lane) were boiled for 5 min and electrophoresed in 12.5% polyacrylamide gels. Separated proteins were transferred onto a nylon membrane and blocked with 5% skimmed milk in PBS containing 0.1% Tween-20 (PBS-T) for 1 h at room temperature. Immunodetection was performed overnight at 4°C in PBS-T using anticaspase-8, anticaspase-9 and anti-Bid IgG. After incubation with an HRP-conjugated secondary IgG (Amersham, London, UK), the blots were developed using ECL (Pierce, Rockford, IL, USA). Protein loading was corrected for *β*-actin.

### Caspase activity assay

After *α*-TOS (30 *μ*M) and/or hrTRAIL (50 ng ml^−1^) treatment, cells were harvested at different time points and lysed in the caspase lysis buffer (1% Nonidet, 5 mM DTT, 150 mM NaCl, 2 mM EDTA, 50 mM Tris-HCl, pH 7.4) containing a cocktail of protease inhibitors. The cytosolic fraction (10 *μ*g protein) was incubated at 37°C in the caspase reaction buffer (1% Nonidet, 100 mM NaCl, 10 mM DTT, 0.1 mM EDTA, 50 mM HEPES, pH 7.5, and 10% glycerol), containing the specific colorimetric substrates for caspases, Ac-IETD-*p*NA for caspase-8 or Ac-LEHD-*p*NA for caspase-9, in a total volume of 100 *μ*l. Caspase activity was evaluated as cleavage of the substrate and monitored at 405 nm in an ELISA plate reader. Caspase activity rates were expressed as optical density (OD) per min, and normalised for protein.

### TRAIL receptor analysis

Expression of TRAIL receptors (DR4, DR5, DcR1, DcR2) was evaluated by flow cytometry before and after treatment with *α*-TOS (30 *μ*M). MM and Met-5A cells were seeded 24 h before the treatment in six-well plates at 3 × 10^5^ per well. HeLa cells were used as a positive control. After 16 h of treatment, floating and attached cells were collected, washed twice with PBS, and incubated at 4°C with antibodies against DR4, DR5, DcR1, and DcR2, followed by a secondary FITC-conjugated IgG, and the cells analysed by flow cytometry. Cytoplasmic expression of TRAIL receptors was assessed after cell permeabilisation. Briefly, cells were fixed in 4% formaldehyde in PBS for 30 min, washed, permeabilised with a saponine solution (0.02% saponine in PBS plus 1% FCS) for 30 min, incubated with the antibodies as described above, and assessed by flow cytometry.

### RT–PCR analysis

Total RNA was isolated from 10^6^ MM and Met-5A cells before and after treatment with 30 *μ*M *α*-TOS, using Trizol (Life Technologies, Rockville, MD, USA), according to the manufacturer's protocol. The first-strand cDNA was synthesised using the GeneAmp RNA PCR kit (PerkinElmer Life Sciences, Boston, MA, USA). PCR analyses were performed in the final volume of 20 *μ*l of buffer containing 1 *μ*l of the retro-transcription product, all four dNTPs (150 *μ*M each), MgCl_2_ (2 mM), 1 U of Taq Gold polymerase (Roche Molecular Biochemicals, Basel, Switzerland), and each primer at 1 *μ*M. The house-keeping gene *β*-actin was used as a loading control. The sequences of primers were as published elsewhere (Bernard *et al*, 2001).

### Immunocytochemistry

The MM cells were placed overnight in 35-mm dishes on poly-L-lysine-coated glass coverslip. After 4 h of incubation with *α*-TOS (30 *μ*M) or hrTRAIL (50 ng ml^−1^) separately or in combination, the cells were washed two times with PBS, fixed with freshly prepared 3% formaldehyde in PBS, and incubated with saponine solution (0.05% saponine and 2% FCS in PBS). Cells were then incubated with mouse antihuman cytochrome *c* IgG in saponine solution for 1 h at room temperature. FITC-conjugated, antimouse secondary IgG was added. The coverslips were mounted on glass slides with Vectashield (Vector Laboratories, Inc., Burlingame, CA 94010, USA) and viewed in a fluorescence microscope.

### Confocal microscope analysis

Met-5A and MM-B1 cells were plated in a six-well plate at 2 × 10^5^ and transiently transfected with pDsRed1-N1 harbouring RFP, and with GFP-expressing vector (pGFP) (BD Biosciences, Palo Alto, CA, USA), respectively. The transfection was carried out by using the GenePorter transfection reagent (Gene Therapy Systems, Inc., San Diego, CA, USA) following the manufacturer's instructions. Briefly, cells were incubated with 1 ml of the serum-free medium DNA/GenePorter transfection reagent complex, which was prepared by mixing 2 *μ*g DNA with 0.5 ml serum-free medium, and this was combined with 10 *μ*l of GenePorter transfection reagent mixed with 0.5 ml serum-free medium, followed by 30 min incubation at room temperature. After 3 h of incubation with the transfection complex, cells were washed and cultured for 48 h. The Met-5A-RFP and MM-B1-GFP cells were mixed and adhered on poly-L-lysine-coated glass coverslips. The transfected Met-5A-RFP and MM-B1-GFP cells were treated for 12 h with hrTRAIL and *α*-TOS at 50 ng ml^−1^ and 30 *μ*M, respectively. The cells were washed two times with PBS, fixed with freshly prepared 3% formaldehyde in PBS, and the coverslip was mounted with Vectashield plus DAPI (Vector Laboratories, Burlingame, CA 94010, USA) and viewed in a confocal microscope (BioRad, MRC 1024, Hercules, CA, USA).

### Statistical analysis

All experiments were conducted at least three times, and data are shown as mean±s.d. Significance was evaluated by the ANOVA repeated measure test. Data were considered statistically significant at *P*<0.05.

## RESULTS

### *α*-Tocopheryl succinate and TRAIL exert different toxicity towards MM and nonmalignant mesothelial cells

**Figure 1:**
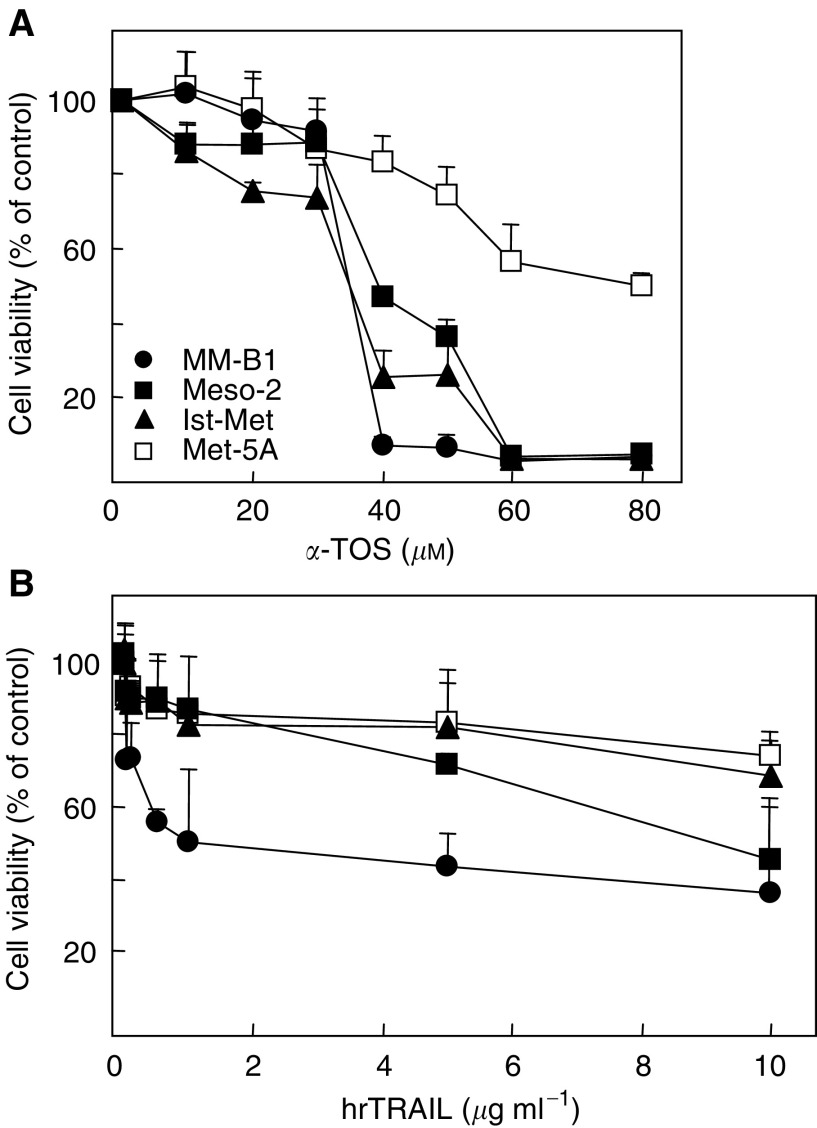
Cytotoxic effect of *α*-TOS and hrTRAIL in MM and mesothelial cell lines. Cells were seeded into 96-well tissue culture plates (2.5 × 10^3^ per well) and treated with increasing concentrations of *α*-TOS (**A**) and hrTRAIL (**B**). Survival curves for MM-B1, Meso-2, Ist-Mes, and Met-5A cell lines are based on the results of MTT assays after 24 h of drug exposure. All data are expressed as mean±s.d. from three independent experiments.

Treatment of MM and nonmalignant mesothelial cell lines with *α*-TOS results in dose-dependent cytotoxicity. Figure 1A

shows viability curves of MM-B1, Meso-2, and Ist-Mes cell lines with IC_50_ values ranging from 34 to 38 *μ*M of *α*-TOS (Table 1

**Table 1:** IC_50_ values and killing efficacy of *α*-TOS, hrTRAIL, and hrTRAIL plus *α*-TOS in malignant mesothelioma and nonmalignant mesothelial cell lines

	***α*-TOS**		**hrTRAIL**		**hrTRAIL**_***α*-TOS**_	
**Cell type**	**IC_50_ (*μ*M)**	**Max effect (% cell death)**	**IC_50_ (*μ*g ml^−1^)**	**Max effect (% cell death)**	**IC_50_ (*μ*g ml^−1^)**	**Max effect (% cell death)**
MM-B1	36±4	96±3	0.3±0.1	64±4	0.010±0.002	87±9
Meso-2	38±7	95±5	5.0±1.0	55±2	0.030±0.004	76±7
Ist-Mes	35±6	96±4	0.7±0.1	32±5	0.100±0.050	69±10
Met-5A	55±8	44±2	0.5±0.1	26±3	0.450±0.150	34±8

The IC_50_ values for *α*-TOS, hrTRAIL, and hrTRAIL (50 ng ml^−1^) plus *α*-TOS (30 *μ*M) were calculated from dose–response curves of MM and nonmalignant mesothelial cells at 24 h of treatment. Data are expressed as mean±s.d. from three independent experiments.

). The cells were relatively resistant to TRAIL-induced cell death; the viability curve reached a plateau at 0.5 *μ*g ml^−1^, and, except for the MM-B1 cells with 40–50% of dead cells, MM cells did not exert more than 10–15% cell death at pharmacologically relevant doses of TRAIL (Figure 1B, Table 1). Importantly, nonmalignant mesothelial cells (Met-5A) exhibited minimal toxicity in the presence of *α*-TOS as well as hrTRAIL at concentrations of up to 80 and 10 *μ*g ml^−1^, respectively (Figure 1A and B).

### *α*-Tocopheryl succinate and TRAIL show synergistic effect towards MM but antagonise each other in nonmalignant mesothelial cells

**Figure 2:**
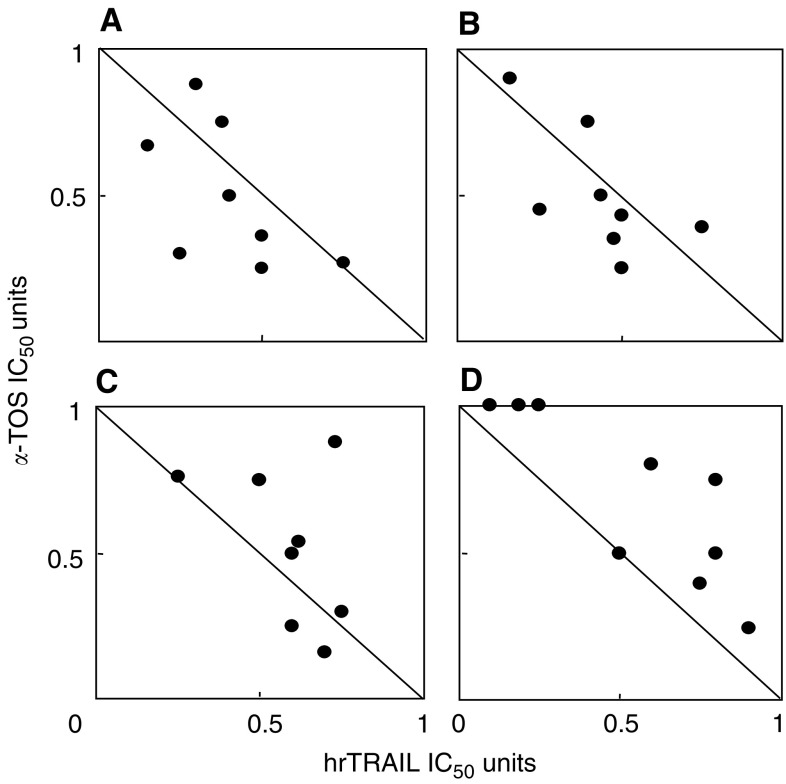
hrTRAIL and *α*-TOS combination exhibit synergistic effects in MM cells and antagonise each other in nonmalignant mesothelial cells. The synergist/antagonistic effect of hrTRAIL and *α*-TOS was evaluated by isobolograms at IC_50_ based on the results of MTT assays for MM-B1 (**A**), Meso-2 (**B**), Ist-Mes (**C**), and Met-5A cells (**D**) treated with combinations of the two drugs. Each diagram combines the results at least of three independent experiments.

**Figure 3:**
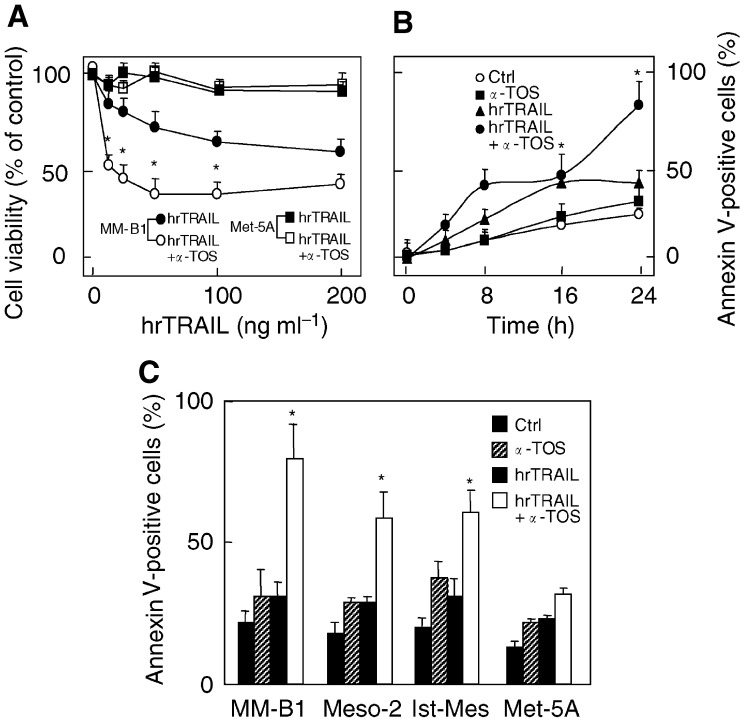
Effect of hrTRAIL, *α*-TOS alone or their combination on apoptosis induction in MM cells. (**A**) MM-B1 and Met-5A cells were exposed to increasing concentrations of hrTRAIL alone (50 ng ml^−1^) or in combination with a sublethal dose of *α*-TOS (30 *μ*M) and their viability was assessed at 24 h. (**B**) MM-B1 cells were exposed to hrTRAIL (50 ng ml^−1^) or *α*-TOS (30 *μ*M) alone or in combination at the indicated time points, and apoptosis was evaluated by annexin-V-FITC. (**C**) Mesothelioma (MM-B1, Meso-2, Ist-Mes) and mesothelial (Met-5A) cells were treated for 24 h with 50 ng ml^−1^ hrTRAIL, 30 *μ*M *α*-TOS, or 50 ng ml^−1^ hrTRAIL plus 30 *μ*M *α*-TOS and evaluated for apoptosis. ^*^ The combined effect was significantly greater than the effects of individual agents, *P*<0.05. Results are expressed as mean±s.d. from three independent experiments.

**Figure 4:**
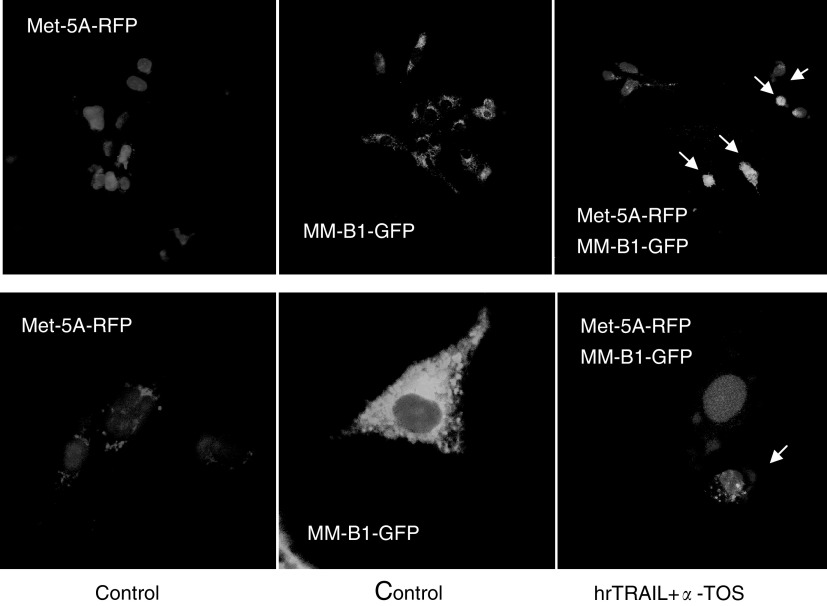
hrTRAIL and *α*-TOS combination selectively induce apoptosis in MM cells. Met-5A and MM-B1 were transiently transfected with pDsRed1-N1 and pGFP, respectively. The transfected cells were combined and allowed to adhere to coverslips, and were treated for 12 h with hrTRAIL and *α*-TOS at 50 ng ml^−1^ and 30 *μ*M, respectively. The coverslips were then mounted in Vectashield plus DAPI and viewed in a confocal microscope. The hrTRAIL/*α*-TOS treatment induced nuclear condensation in the ‘green’ MM-B1-GFP cells (arrows), whereas no such nuclear morphological changes were observed in the ‘red’ Met-5A-RFP cells.

To investigate the effect of *α*-TOS on TRAIL-induced cytoxicity, MM cells were exposed to increasing concentrations of individual drugs alone and in combination, cell viability was assessed and isobologram analysis performed. Isobolograms were created at the IC_50_ values as described elsewhere (Tsai *et al*, 1989). The IC_50_ unit values for *α*-TOS <1 were plotted against corresponding IC_50_ unit values for hrTRAIL; the distribution of points along the diagonal connecting the values of 1 suggests an additive effect of the two drugs while the points below or above the line indicate their synergism and antagonism, respectively (Figure 2

). Thus, the effects of *α*-TOS and hrTRAIL on cell death induction were synergistic in MM-B1 and Meso-2 cells and, to a lesser extent, in Ist-Mes cells where a synergistic/additive effect was observed. Notably, an antagonistic effect was found when the two drugs were combined in nonmalignant mesothelial cells, in which most of the points lie above the diagonal. Table 1 shows that the high IC_50_ values for TRAIL decreased by a factor of ∼10–100 when *α*-TOS was present at subapoptotic levels in the case of MM cells, while no effect was observed for Met-5A cells. Finally, *α*-TOS enhanced TRAIL-induced apoptosis of MM cells in a dose- and time-dependent manner, while only marginal and nonsignificant apoptosis was observed in the nonmalignant mesothelial cells (Figure 3

). We next attempted to directly show the selectivity of MM cells for synergistic effect of TRAIL and *α*-TOS. For this, we transfected the Met-5A cells with a plasmid harbouring RFP, while MM-B1 cells with a GFP. The cells were then combined in culture and exposed to hrTRAIL plus *α*-TOS. Figure 4

reveals that the combination of the two agents selectively induced apoptosis MM cells without affecting the nonmalignant mesothelial cells, since the DAPI-visualised nuclei were condensed in the ‘green’ malignant cells, whereas no nuclear morphological changes were observed in the ‘red’ nonmalignant cells (64±8 *vs* 10±4% of apoptotic cells for MM-B1 and Met-5A, respectively). These observations directly document the intriguing paradigm of selective sensitisation of MM cells to TRAIL-dependent killing by *α*-TOS.

### *α*-Tocopheryl succinate differentially modulates expression of TRAIL receptors

**Figure 5:**
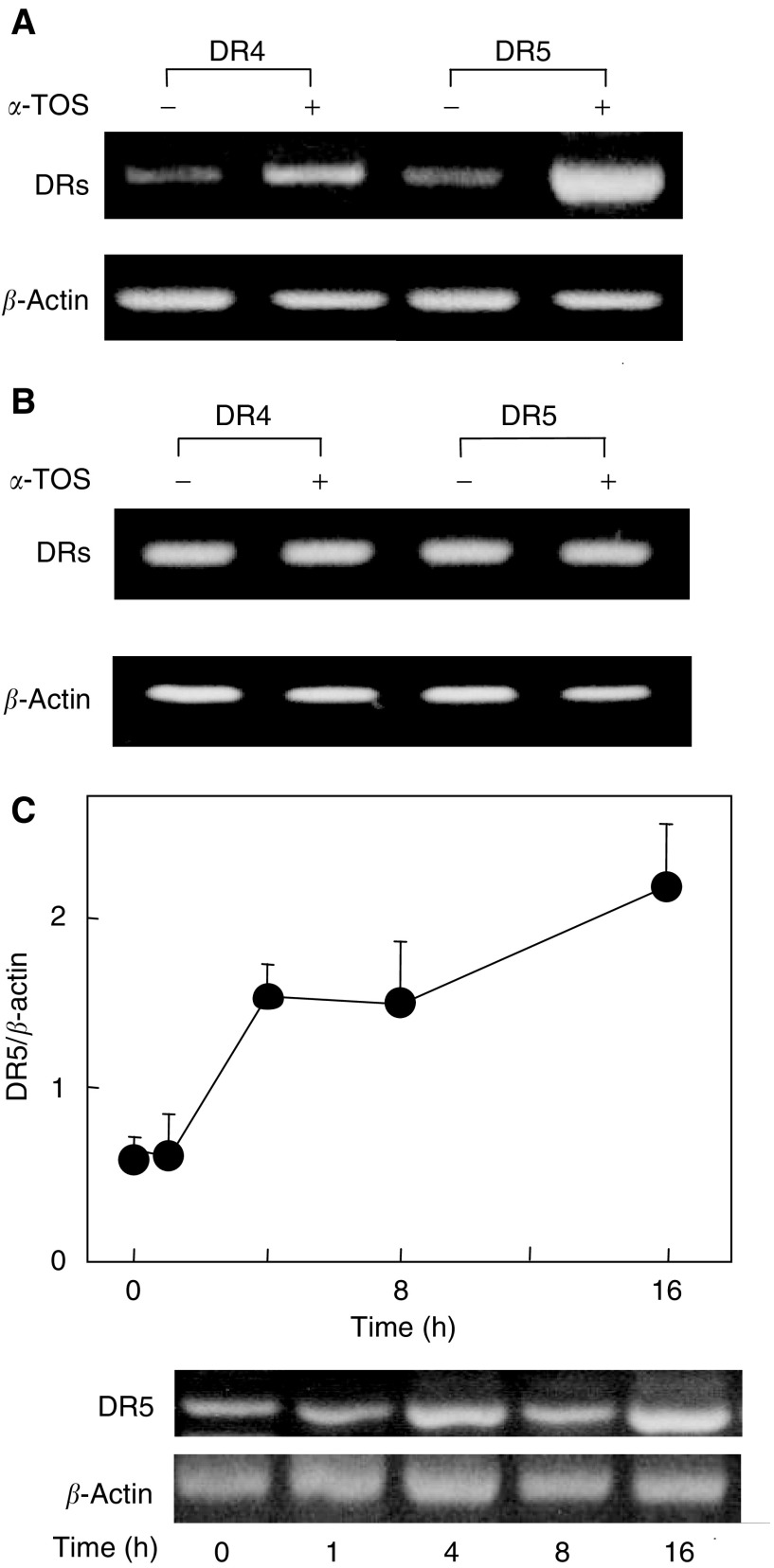
Effect of *α*-TOS treatment on the expression of TRAIL receptors in MM and mesothelial cells. The death receptors (DR4, DR5) were evaluated in MM-B1 (**A**) and Met-5A cells (**B**) as mRNA expression before and after treatment with 30 *μ*M *α*-TOS for 4 h. (**C**) MM-B1 cells were treated with 30 *μ*M *α*-TOS at the indicated time points, and mRNA expression of DR5 evaluated. Results in panels (**A**)–(**C**) are representative of three independent experiments.

**Figure 6:**
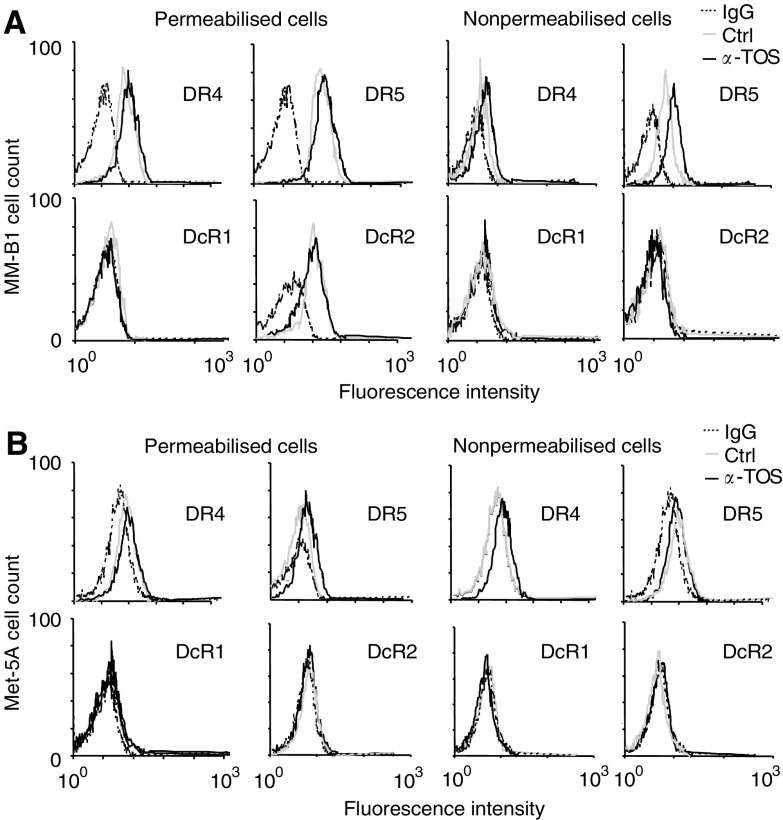
Cell surface expression of TRAIL receptors evaluated on permeabilised and nonpermeabilised MM and mesothelial cells after *α*-TOS treatment. TRAIL receptor protein analysis was performed on MM-B1 (**A**) and Met-5A (**B**) cells. Cells were seeded at 3 × 10^5^ per well into six-well plates and exposed to *α*-TOS for 16 h. Permeabilised and nonpermeabilised cells were then incubated with antibodies against DR4, DR5, DcR1, or DcR2, and their expression was evaluated by flow cytometry. Results are representative of three independent experiments with similar results.

To explore the possibility that *α*-TOS modulates TRAIL cytotoxicity by regulating expression of its receptors, we analysed the extent of TRAIL death and DcR expression at both mRNA and protein level. RT–PCR analysis shows that the expression of the DR4 and, in particular, DR5 mRNA was enhanced in MM cells treated with subapoptotic (30 *μ*M) *α*-TOS (Figure 5A

). On the other hand, the level of the expression of the DcR1 and DcR2 mRNA remained unchanged (data not shown). *α*-TOS failed to modulate the level of expression of both death and DcR mRNA in Met-5A cells (Figure 5B). Time course analysis shows that exposure of MM cells to *α*-TOS upregulated DR5 in a time-dependent manner, with a two-fold increase in DR5 mRNA at 4 h (Figure 5C), that is, at least 4 h before the detection of first signs of apoptosis (cf Figure 3B). Flow cytometric analysis, performed on both nonpermeabilised and permeabilised cells revealed that MM cells express both DR4 and DR5 protein on cell surface and in the cytoplasm (Figure 6A

). DcR1 was not expressed in any of the cell lines tested, whereas DcR2 was detected only in the cytoplasmic compartment (Figure 6A). Sublethal doses of *α*-TOS increased the expression of the DR4 and, more so, DR5 protein without affecting expression of the DcR1 and DcR2 protein (Figure 6A). The basal level of the DR4 and DR5 protein was lower in Met-5A cells then in their malignant counterparts, whereas no expression of the DcR1 and DcR2 protein was detected in Met-5A cells (Table 2

**Table 2:** Evaluation of cell surface expression of TRAIL receptors before and after *α*-TOS treatment in MM and mesothelial cells

	**DR4**		**DR5**		**DcR1**		**DcR2**	
**Cell type**	**Ctrl**	***α*-TOS**	**Ctrl**	***α*-TOS**	**Ctrl**	***α*-TOS**	**Ctrl**	***α*-TOS**
MM-B1	2.5±0.30	3.1±0.25	4.3±0.50	**5.9±0.72**	1.2±0.20	1.2±0.21	1.0±0.11	1.0±0.14
Meso-2	1.6±0.25	**2.4±0.32**	2.0±0.12	**6.7±0.52**	1.1±0.21	1.1±0.35	1.0±0.10	1.0±0.12
Ist-Mes	1.7±0.54	2.0±0.38	3.6±0.61	4.3±0.52	1.3±0.20	1.1±0.22	1.0±0.11	1.0±0.09
Met-5A	1.9±0.31	2.2±0.41	1.7±0.46	1.8±0.33	1.0±0.05	1.0±0.06	1.0±0.14	1.2±0.08

The expression of TRAIL receptors was evaluated by flow cytometry before ***(Ctrl)*** and after treatment with *α*-TOS (30 *μ*M, 16 h) in MM and mesothelial cells. The values represent the fluorescence intensity of the receptors normalised for a blank incubated with an irrelevant primary antibody. Data are expressed as mean±s.d. from three independent experiments and statistical differences between controls *vs* *α*-TOS treatment are bold, *P*<0.005.

). Finally, Met-5A cells exposed to *α*-TOS showed only a nonsignificant increase of the DR expression (Figure 6B, Table 2).

### *α*-Tocopheryl succinate facilitates the TRAIL proapoptotic signalling

**Figure 7:**
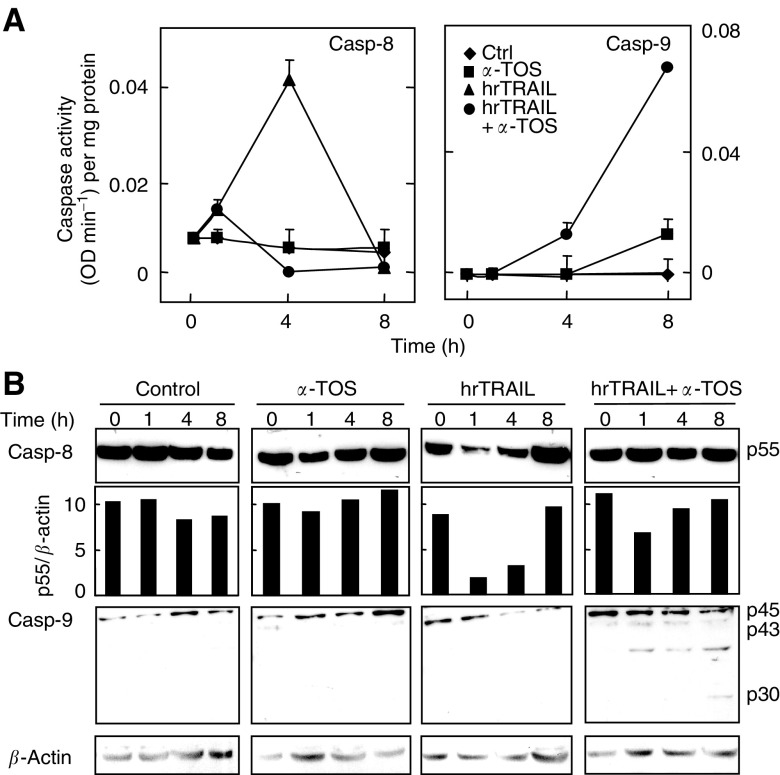
Activation of caspase-8 and -9 by hrTRAIL and *α*-TOS alone or by their combination in MM cells. MM-B1 cells were treated with 50 ng ml^−1^ hrTRAIL, 30 *μ*M *α*-TOS, or 50 ng ml^−1^ hrTRAIL plus 30 *μ*M *α*-TOS, the activity of caspase-8 and -9, expressed as OD/min per mg protein (**A**), or caspase-8 and -9 cleavage (**B**), were assessed at the indicated time points. Data are expressed as mean±s.d. from three independent experiments.

TRAIL-induced apoptosis can be characterised by activation of caspases, including caspase-8, -9, and -3, with their pattern of activation being cell type-dependent (Griffith *et al*, 1998). When MM cells were treated with TRAIL, caspase-8 was transiently activated, as shown by increased protease activity at 4 h and by the procaspase cleavage as soon as at 1 h of incubation (Figure 7A and B

). This activation of caspase-8 at 4 h was correlated with only low levels of apoptosis (cf Figure 3B and C). Combined treatment with TRAIL and *α*-TOS, which overcame resistance of MM cells to TRAIL, resulted in very low level of caspase-8 activity, but promoted caspase-9 activation (Figure 7A and B).

**Figure 8:**
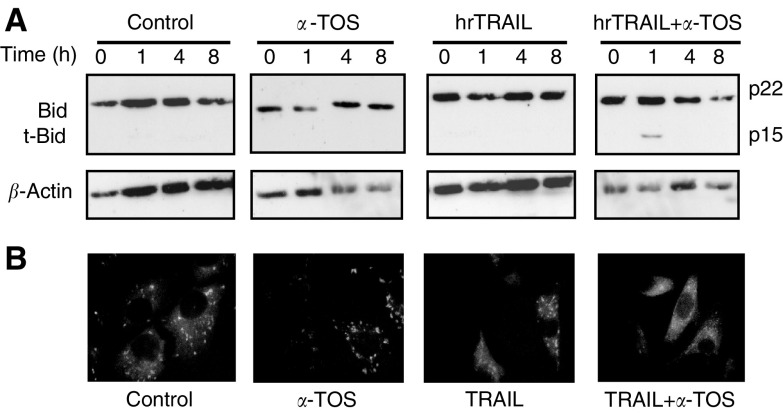
Drug combination determines Bid cleavage and cytochrome *c* release. MM-B1 cells were treated with 50 ng ml^−1^ hrTRAIL, 30 *μ*M *α*-TOS, or 50 ng ml^−1^ hrTRAIL plus 30 *μ*M *α*-TOS, Bid cleavage (**A**) and cytochrome *c* released from mitochondria (**B**) were assessed. Picture shown is representative for at least two independent experiments.

**Figure 9:**
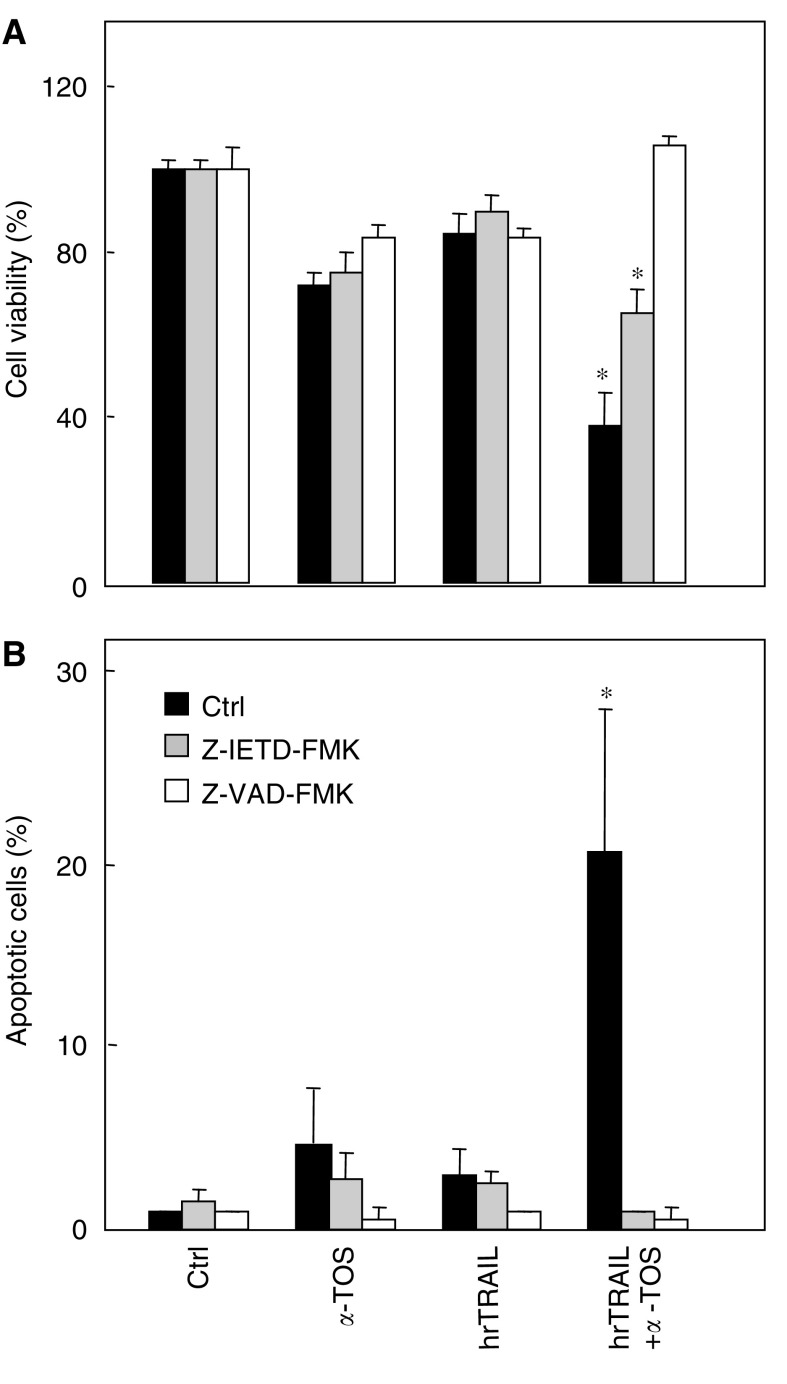
Caspase inhibitors suppress the synergism of hrTRAIL and *α*-TOS. MM-B1 cells were treated with 50 ng ml^−1^ hr-TRAIL, 30 *μ*M *α*-TOS, or 50 ng ml^−1^ hr-TRAIL plus 30 *μ*M *α*-TOS, in the absence or presence of the caspase-8 inhibitor Z-IETD-FMK or the pan-caspase inhibitor Z-VAD-FMK, and evaluated for cell viability by MTT analysis (**A**) or apoptosis by the comet assay (**B**). The symbol ‘^*^’ indicates statistical differences (*P*<0.05) in the values for cells treated with *α*-TOS and hrTRAIL alone and those co-treated with the two agents. Data are expressed as mean±s.d. from three independent experiments.

The link between caspase-8 and -9 activation may be mediated by the proapoptotic cleavage of Bid. Indeed, combination of *α*-TOS and TRAIL resulted in early (1-h) Bid cleavage, triggering the ensuing proapoptotic mitochondrial events, including the cytosolic mobilisation of cytochrome *c* (Figure 8A and B

). Preincubation with the caspase-8 inhibitor Z-IETD-FMK inhibited the synergistic effect of TRAIL and *α*-TOS but not *α*-TOS-induced apoptosis, while treatment with the pan-caspase inhibitor, Z-VAD-FMK, completely inhibited apoptosis in MM cells, regardless of the treatment regimen (Figure 9A and B

).

## DISCUSSION

The aim of the study was to investigate the effect of combined treatment of MM cells with *α*-TOS and TRAIL. As *α*-TOS exerts its proapoptotic activity via the distal, mitochondrial route, and TRAIL-induced cell death is relayed via the proximal pathway, triggered by the cell surface receptor engagement, we hypothesised that the two different modes of action could potentiate apoptosis in the TRAIL-resistant MM cells. If so, TRAIL and the vitamin E analogue could represent interesting drugs for combinatorial treatment of MM, a thus far incurable neoplastic disease.

Our results herein clearly demonstrate a cross-talk between *α*-TOS and TRAIL in several processes resulting in efficient apoptosis induction in MM cells. Thus, in all MM cell lines tested, *α*-TOS was found to enhance TRAIL-induced cell death in a synergistic manner regardless of their phenotype (*cf* Figure 2 and 3), while MM cells exerted relative resistance to TRAIL alone at as much as 10 *μ*g ml^−1^ of the agent. Important too, *α*-TOS and TRAIL exerted an antagonistic effect in case of nonmalignant mesothelial cells (*cf* Figure 2). This is a highly intriguing finding, since it points to the possibility that, if applied together, *α*-TOS and TRAIL will efficiently and selectively kill MM cells while protecting the neighbouring nonmalignant mesothelial cells of the pleural or peritoneal cavity (*cf* Figure 4). This notion is consistent with reports showing protective activity of *α*-TOS towards normal cells and tissues (Vuchetich *et al*, 1996; Zhang *et al*, 2001). The reason for this protective effect is, most likely, due to conversion of *α*-TOS to *α*-tocopherol (*α*-TOH), as shown, for example, for hepatocytes (Fariss *et al*, 2001). This is consistent with the notion that *α*-TOS has a dual activity: as *α*-TOS, the provitamin form, it causes apoptosis in malignant cells, thereby suppressing tumour growth, while as *α*-TOH, the vitamin form, it acts as an antioxidant protecting normal cells from insults such as inflammation (Neuzil, 2002).

The biphasic viability curve for MM cells treated with TRAIL (*cf* Figure 1B) indicates either the presence of two different cell populations, resistant and sensitive, or the development of resistance during treatment. Indeed, MM cells pretreated with TRAIL developed resistance against a second TRAIL exposure, reaching a maximum at 8 h and decaying after 24 h of incubation (Tomasetti *et al*, unpublished data), suggesting an intrinsic mechanisms of the cells for evading apoptosis. The presence of this resistant subpopulation may be due to initial activation of survival pathways (Ehrhardt *et al*, 2003), and *α*-TOS may sensitise the resistant cells by interfering with these pathways (Neuzil, 2002).

A cooperative proapoptotic effect of *α*-TOS with immunological apoptogens has been observed in breast cancer (Yu *et al*, 1999) and colon cells (Weber *et al*, 2002). The former report showed that *α*-TOS converted the Fas-resistant to Fas-sensitive cells via mobilisation of the Fas receptor from the cytosol to the plasma membrane. Thus, enhancement of DR expression on cell surface could enhance cancer surveillance. We show here that higher expression of TRAIL DRs, in particular DR5, by *α*-TOS on the surface of MM cells was due to *de novo* synthesis rather than translocation of TRAIL receptors from cytoplasm to the plasma membrane (*cf* Figure 6). It has been reported previously that cells can be sensitised to TRAIL by upregulation of DR5 in response to activation of the transcription factor p53, suggesting that enhanced DR5 expression may be a common mechanism for apoptosis induction by DNA-damaging agents (Wu *et al*, 1997). We have recently observed that exposure of cancer cells to *α*-TOS results in early generation of reactive oxygen species (ROS) (Weber *et al*, 2003), and we have also observed phosphorylation of p53 (Tomasetti *et al*, unpublished data). It is thus feasible that a p53-mediated process contributes to *α*-TOS-induced DR5 upregulation in MM cells, consistent with another report suggesting p53 activation as a mode of sensitisation of cancer cells towards TRAIL killing (Xie *et al*, 2001). Although Met-5A cells, used here as a control cell line, are immortalised with SV-40 resulting in inactivation of p53, translocation of p53 into the nucleus was also observed in these cells following *α*-TOS treatment (unpublished data). However, p53 activation results in a small increase of DRs expression in Met-5A, contrary to that observed in MM cell lines. Since we have also found that exposure of cells to *α*-TOS leads to very fast activation of sphingomyelinase resulting in early accumulation of ceramide (Weber *et al*, 2003), which has been suggested to upregulate DRs (Herr *et al*, 1999), additional/alternative mechanisms cannot be ruled out, including modulation of expression of bcl-2 family proteins (Ray and Almasan, 2003). However, we observed that upregulation of DRs is not followed by an increase of caspase-8 activity suggesting that other mechanisms are involved in the synergistic *α*-TOS/TRAIL MM cell killing.

Kinetics analysis of TRAIL-induced signalling revealed a transient activation of caspase-8, which resulted in induction, albeit low, of apoptosis. Caspase-8 activation was less pronounced in the presence of TRAIL plus *α*-TOS. Under this setting, we observed activation of the mitochondria-dependent apoptotic pathway, including Bid cleavage, cytochrome *c* cytosolic mobilisation and, finally, caspase-9 activation. Bid cleavage may lead to mitochondrial translocation of Bax, as shown for *α*-TOS in other cancer models (Weber *et al*, 2003; Yu *et al*, 2003). Preincubation of MM cells with the caspase-8 inhibitor Z-IETD-FMK inhibited the synergistic effect of TRAIL and *α*-TOS, while treatment with the pan-caspase inhibitor Z-VAD-FMK completely inhibited apoptosis initiated by the two inducers alone or in combination. These results clearly document that caspase-8 activation is essential for the TRAIL-dependent effect, and that this is significantly amplified by activation of caspase-9 in the presence of subapoptotic levels of *α*-TOS. Thus, there is a cross-talk between *α*-TOS and TRAIL in potentiation of apoptotis in the TRAIL-resistant MM cells, in particular in linking the receptor- and mitochondria-associated events.

Our data suggest that cleavage of Bid may be considered as the link between the proximal and distal apoptotic signalling pathways. A similar scenario for a cross-talk between the two routes has been suggested by others. Thus, cleavage of Bid is involved in sensitisation to TRAIL of colorectal (Lacour *et al*, 2001; Ravi and Bedi, 2002) and prostate cancer cells (Nimmanapalli *et al*, 2001). These reports show an increased activity of caspase-8 associated with the sensitisation, suggesting the type II cells, that is, cells where receptor-mediated proapoptotic signalling is transmitted downstream via Bid cleavage following the death-inducing signalling complex (DISC) formation on the cytosolic terminus of the DRs (Ashkenazi and Dixit, 1998). However, our observations with MM cells point to Bid cleavage at minimal caspase-8 activation in the presence of TRAIL and *α*-TOS, while TRAIL alone, causing higher activation of caspase-8, is inefficient in apoptosis induction and Bid cleavage does not occur. Therefore, there appears to be a block in the type I signalling in MM cells downstream from caspase-8 to the effector caspases, and a cross-talk between *α*-TOS and TRAIL, causing Bid cleavage resulting in mitochondrial signalling that then culminates in efficient apoptosis. Thus, MM cells, from the point of view of apoptotic signalling, may represent atypical type II cells (Scaffidi *et al*, 1998), possibly consistent with their general resistance to apoptosis, causing mesotheliomas incurable.

Taken together, we report that sublethal doses of *α*-TOS selectively enhance TRAIL-dependent apoptotic signalling in MM via mitochondrial pathways, while antagonising the activity of TRAIL in nonmalignant mesothelial cells. The observed sensitisation of MM cells to TRAIL killing is associated with increased expression of DR4 and DR5, and with activation of the mitochondrial proapoptotic signalling with Bid cleavage linking the proximal and distal events, although the precise contribution of the parallel/auxiliary pathways to the overall efficacy of apoptosis execution in MM cells is not known at present. The importance of activation of both types of signalling in sensitisation of cancer cells to TRAIL has been recently shown for prostate cancer cells (Ray and Almasan, 2003). Our findings are in accord to those of Liu *et al*, 2001, reporting that sensitisation of MM cells toward TRAIL by chemotherapeutic is dependent on the mitochondrial pathway, in a c-Jun N-terminal kinase (JNK)-dependent manner (Vivo *et al*, 2003). Thus, *α*-TOS, although also acting via the JNK pathway (Yu *et al*, 2001) signalling downstream through Bax (Lei *et al*, 2002), promotes the receptor-dependent pathway, thereby maximizing the apoptotic potential of the cell. In conclusion, our data show that *α*-TOS efficiently potentiates TRAIL killing of MM cells that are highly resistant to established treatment via mitochondrial mechanisms, and prompt testing of TRAIL and *α*-TOS, as well as other vitamin E analogues (Birringer *et al*, 2003), in preclinical models of MM. The potential combinatorial clinical use of *α*-TOS and TRAIL is supported by our recent findings that the vitamin E analogue promotes survival of immunocompromised mice with experimental peritoneal mesothelioma (Tomasetti *et al*, in press).
